# Effects of Field Fumigation and Inoculation With the Pecan Truffle (*Tuber lyonii*) on the Fungal Community of Pecan (*Carya illinoinensis*) Seedlings Over 5 Years

**DOI:** 10.3389/fmicb.2021.661515

**Published:** 2021-05-13

**Authors:** Arthur C. Grupe, Michelle A. Jusino, Alija B. Mujic, Brantlee Spakes-Richter, Gregory Bonito, Tim Brenneman, Matthew E. Smith

**Affiliations:** ^1^Department of Plant Pathology, University of Florida, Gainesville, FL, United States; ^2^Department of Biology, William & Mary, Williamsburg, VA, United States; ^3^Department of Biology, California State University, Fresno, Fresno, CA, United States; ^4^Department of Plant, Soil and Microbial Sciences, Michigan State University, East Lansing, MI, United States; ^5^Department of Plant Pathology, University of Georgia, Tifton, GA, United States

**Keywords:** agroecology, ectomycorrhizae, field inoculation, pecan truffle, truffle orchard

## Abstract

Truffle fungi are esteemed for their aromatic qualities and are among the most widely cultivated edible ectomycorrhizal fungi. Here we document a successful method for establishing *Tuber lyonii*, the pecan truffle, on pecan (*Carya illinoinensis*) seedlings in a field setting. We assessed the impacts of soil fumigation and varying concentrations of truffle spore inoculum on the ectomycorrhizal fungal and the complete fungal communities as well as the colonization of *T. lyonii* on pecan roots at three nurseries in Georgia, United States. To identify fungal communities on pecan seedlings, we performed high-throughput amplicon sequencing of the fungal ITS1 rDNA region. Our 5-year long field experiment demonstrates that fumigation and inoculation together resulted in the highest persistence of *T. lyonii* on pecan roots. While fungal OTU numbers fluctuated over the years of our experiments, there was no statistical support to demonstrate diversification of communities when Shannon diversity metrics were used. However, we did find that older seedlings were less likely to be dominated by *T. lyonii* compared to younger ones, suggesting successional changes in the fungal community over time. This suggests that transplanting inoculated seedlings after 2 or 3 years post-inoculation is optimal for future truffle propagation efforts. Our results demonstrate that *T. lyonii* can be established *in situ* with methods that are compatible with current pecan nursery industry practices and that fungal communities on pecan seedlings vary depending on the experimental treatments used during planting. While the pecan truffle is not yet widely cultivated, our results provide insights for future large-scale cultivation of this and perhaps other *Tuber* species.

## Introduction

Truffle fungi are characterized by closed sporocarps in which the fertile, spore-bearing layer is totally encased by sterile tissue at maturity. Forcible spore discharge has been lost in most truffle lineages, which instead rely upon animal mycophagy or other methods for dispersing their spores (Luoma et al., 2003; Trappe et al., 2009; Elliott et al., 2019; Vašutová et al., 2019). The use of animals for dispersal is facilitated by volatile organic compounds (VOCs) released by truffle fruiting bodies through the activity of endogenous bacterial communities at maturity (Splivallo et al., 2015). Truffles in the genus *Tuber* (Tuberaceae, Ascomycota), release VOCs that are gastronomically enticing and may mimic animal pheromones (Claus et al., 1981; Splivallo et al., 2011). Members of the genus *Tuber* grow in a mutually beneficial symbiotic relationship, known as ectomycorrhizae (ECM), with the roots of certain host trees (Bonito et al., 2010; Tedersoo and Smith, 2013). The fungus receives carbohydrates from the host and in exchange, the mycobiont provides greater access to water, essential nutrients, added protection from fungal root pathogens and stress from drought (Smith and Read, 2008).

Truffle fungi play important roles in both ecosystems and local economies. Given the high culinary value of select *Tuber* spp., truffle cultivation promises unique opportunities in sustainable agriculture. For example, studies in Europe have shown that establishing truffle orchards by planting truffle-inoculated trees can be an incentive to reforest fallow land, non-arable land, and hillsides that had previously been cleared for timber or agriculture (Bonet et al., 2006; Samils et al., 2008). Planting truffle orchards can stimulate rural economies, as truffle harvests produce an excellent source of income, although there is a significant delay between the time that the truffle orchard is initiated and the time that truffles are harvested (Samils et al., 2008). Among the most important factors that determine whether the truffle orchard will fail or will be successful include: the quality of the inoculated seedlings, the amount of colonization by the selected *Tuber* species on the seedlings in the nursery, matching soil and environmental conditions that favor both the truffle and the tree host, and control of fungal contaminants in the nursery (Olivier et al., 2002; Samils et al., 2008; Murat, 2015). However, the production processes prior to sale of the seedlings, such as inoculum identification, growing of seedlings in greenhouses, and assessing seedlings for *Tuber* colonization, increase the cost when compared to non-inoculated seedlings (Bonet et al., 2009). While the initial cost to set up a truffle orchard is high, planting economically productive nut trees that have also been inoculated with *Tuber* spp. has the potential to increase the long-term income from these orchards because a second revenue stream can be generated from the same land.

Recent studies have determined that pecan (*Carya illinoinensis*) is a compatible host for truffles and forms ectomycorrhizal associations with many commercial *Tuber* species, including native and Eurasian species (Bonito et al., 2011; Benucci et al., 2012; Ge et al., 2017; Marozzi et al., 2017). Thus, pecan trees are an attractive host that could be used for co-cropping of truffles in the southeastern United States, where pecans are a major agricultural crop. The state of Georgia is the top producer of pecans and currently has approximately 6.5 million hectares of pecan trees (Wells, 2018). Pecan trees are also commonly associated with a native truffle species, *Tuber lyonii* Butters (the pecan truffle), which is found in pecan orchards across the Southeastern United States (Hanlin et al., 1989; Bonito et al., 2011; Ge et al., 2017). While the exact suite of factors responsible for the presence and persistence of pecan truffles in orchards is unclear, Ge et al. (2017) found a strong correlation between soil pH and the presence of *Tuber* species. In their study orchards with a higher soil pH were more likely to be dominated by *Tuber* and other Pezizales ECM. Although *T. lyonii* lacks the culinary reputation of European *Tuber* species, it has a desirable flavor that is savory and nutty. With a price range of $200–350 USD per pound, this truffle provides an affordable gourmet experience for consumers and a potential profit incentive for growers. The profit margin is, however, lower than for commonly cultivated European truffles (e.g., *Tuber aestivum* and *Tuber melanosporum*), and the cost of seedling production would need to be reduced in order to make co-cropping a more feasible option for pecan growers. One way to reduce the cost of providing inoculated pecan seedlings would be to inoculate seedlings *in situ* when establishing commercial nurseries, rather than in greenhouse nurseries, which is typical for the truffle inoculation industry but more costly. *In situ* field inoculation of truffles would allow for large-scale, commercial production of inoculated pecan seedlings, with minimal modification of existing cultural practices. While *in situ* field inoculations are not the historic method of choice, this technique would be better in-line with the agro-economical interests and desires of pecan growers who are primarily interested in pecan production. Therefore, our methods reflect our desire to develop techniques that will work to enhance the co-cropping of the pecan truffle with pecan trees. Optimally, effective protocols would compliment current pecan nursery practices and infrastructure with minimal additional costs and protocols to growers. Paramount to this would be maintaining a viable amount of *T. lyonii* colonized ECM root tips for out-planting, even when seedlings are extracted from the nursery and transplanted to new orchard sites using mechanical extraction techniques.

The typical practice for establishing a commercial pecan nursery involves the soil being deep turned and shaped into raised beds. Most nurseries cover the beds with plastic and then inject a fumigant (methyl bromide and/or chloropicrin) with a shank under each bed to clear the soil of pathogens prior to planting. Drip lines are also installed at this time to provide irrigation and allow for injection of liquid fertilizer. Once fumigants decrease to a safe level, pecan seeds are planted approximately 25 cm apart, grown for 1 year before grafting with scion wood of a selected cultivar, and then finally excavated and sold either 2 or 3 years after initial planting (Brenneman pers. obs.). Rearing truffle-inoculated seedlings in a field nursery, as opposed to greenhouses, could allow for inexpensive production of inoculated seedlings and would be compatible with the current industry model for establishing pecan groves. However, challenges of this co-production system compared to those of a traditional pecan nursery are the: (1) increased costs of obtaining sufficient quality and quantity of truffle inoculum due to the high volume of seedlings, (2) potential invasion of other ECM fungi onto seedlings thereby creating competition that may limit pecan truffle colonization, (3) the additional initial effort and costs of inoculating seedlings with truffles during planting, and (4) loss of mycorrhized root tips during mechanical extraction of transplants from nursery beds. Most commercial pecan nurseries use soil fumigants to reduce problems with weeds, nematodes and soil-borne diseases and their movement across different orchard environments (Brenneman pers. obs.). The potential effects that these soil fumigants, specifically methyl bromide, may have on truffle inoculations and the resulting ECM communities after fumigation have been previously assessed in limited studies (Sourzat et al., 1990) but not for pecans. While there is concern regarding the use of methyl bromide due to its negative environmental impacts, this study focused on utilizing existing and common commercial nursery practices to asses the potential for successful co-cropping of the pecan truffle with pecans. Although methyl bromide will eventually be phased out of pecan seedling production, as it has been for most other crops, it will likely be replaced with another fumigant formulation, retaining fumigation as a standard practice.

Based on preliminary observations in pecan orchards and nurseries we hypothesize that following soil fumigation: (1) pecan seedlings can be inoculated with *T. lyonii* spores to achieve truffle colonization in typical commercial field nurseries, (2) there is a minimum threshold of truffle inoculum needed to ensure that ECM roots are infected by *T. lyonii* while still conserving the amount of inoculum required, and (3) fumigation and inoculation will both strongly influence fungal community composition over time. To investigate our hypotheses, we initiated two concurrent experiments. The first experiment, termed the concentration experiment, investigated how the concentration of truffle inoculum would influence truffle colonization and the fungal communities on pecan seedlings at three farms. A second experiment, termed the fumigation and inoculation block experiment, used a fully randomized, block experiment to elucidate the interplay between fumigation and inoculation and also explore their influence on truffle colonization and the ECM fungal communities on pecan seedlings at one experimental farm.

## Materials and Methods

### Site Locations and Characteristics

Three sites in southern Georgia, United States were selected for these experiments, two commercial pecan nurseries and one experimental nursery. One of the commercial nurseries is located in Screven County (Screven), GA and the other in Berrien County (Berrien), GA. The University of Georgia Ponder experimental orchard is located in Tift County (Tift), GA. To determine baseline soil nutrient characteristics at each site, composite soil samples were taken from each site at the time of the first seedling harvest in year 2 (Table 1). Dried soil samples were submitted to the University of Florida Institute of Food and Agricultural Sciences Extension Soil Testing Laboratory^1^ and analyzed for pH, P, K, Ca, Mg, Zn, Mn, Cu, Cl, and percent organic matter following the methods used by Ge et al. (2017).

**TABLE 1:** Composite soil characteristics where pecan seedlings were planted at one experimental and two commercial pecan nurseries.

**Farm**	**pH**	***P***	**K**	**Ca**	**Mg**	**Zn**	**Mn**	**Cu**	**Cl**	**Organic material (%)**
Tift	6.06	2.8	45.06	17.28	3.86	0	0.19	0	16.73	1.18
Berrien	6.5	2.52	10.69	16.08	5.32	0.02	0.22	0	9.94	0.52
Screven	5.99	4.81	30.91	15.95	4.3	0	0.44	0	9.41	1.24

*Soil measurements were performed during year 2 of the experiment. Units for elements analyzed are in mg/kg.*

### Field Site Preparation and Inoculation

In April 2013, 50 m by 50 m experimental fields were prepared prior to planting in accordance with common nursery practices. Rows were fumigated at a depth of 20 cm through the three injection shanks that were installed per bed with a mixture of methyl bromide and Chloropicrin, a commercially available broad-spectrum biocide. We used a broadcast rate of 158.757 Kg per 0.405 hectare of a 67:33 methyl bromide:Chloropicrin mixture under low-density polyethylene mulch (Eure and Culpepper, 2017). After the fumigant was injected beneath the covering and allowed 24 h to penetrate, the treated soil was allowed to vent for 10–14 days. After venting, cold-stratified pecan seeds (Adams and Thielges, 1978) of the “Desirable” variety were planted in rows, 60 seeds per treatment, 25 cm apart from each other, 1.5 m between treatments within the same row, with a 1.5 m gap between rows, according to treatments outlined below.

Inoculation material consisted of sporocarps of *T. lyonii* that were previously collected in summer and fall of 2012 and frozen at −20°C. Sporocarps were identified as *T. lyonii* in the field by M. Smith and T. Brenneman, and the identity of randomly selected truffles was confirmed via light microscopy and Sanger sequencing of the ITS rDNA using established methods (Bonito et al., 2012). Truffles were soaked in ice water and cleaned by hand with a tap water rinse. Truffles with visible parasites or decay were discarded. A truffle spore slurry was prepared by combining clean, ice-cold truffles with a small amount of ice water and homogenizing them with a commercial blender. This homogenized liquid suspension of truffles was used for inoculation of seedlings following the methods of Bonito et al. (2011) with one exception: we applied a maximum rate of 2 g of homogenized *T. lyonii* in 50 ml of water per seedling. This is in contrast to the treatment of Bonito et al. (2011) which used only 1 g of truffle spores per seedling in sterile soils in a greenhouse setting. Because our inoculations were done in the field under non-sterile conditions, we opted to use a larger volume of truffle spores in our inoculation.

We conducted two different inoculation experiments to investigate whether (1) *T. lyonii* could colonize pecan seedlings in the field, and (2) to determine whether inoculation with *T. lyonii* influences the communities of other ECM fungi in the pecan nursery setting. In the first experiment we investigated how the concentration of truffle inoculum would influence truffle colonization of roots and the ECM fungal communities on pecan seedlings at three sites. A maximum application rate of 2 g of truffle spores per seedling and subsequent dilutions resulted in four additional treatments; 1 g, 0.2 g, 0.1 g, 0.02 g, and a non-inoculated control of 0 g of truffle spores per seedling.

In the second experiment, carried out at the UGA Ponder site (Tift), a fully randomized block experiment was designed to understand the effects of fumigation and inoculation, and their interaction, on truffle colonization and ECM fungi communities on pecan seedlings. Seedlings in the fumigation and inoculation block experiment were each exposed to one of the following four treatments: fumigation and inoculation, fumigation and no inoculation, no fumigation and inoculation, and no fumigation and no inoculation. For all inoculations in the fumigation and inoculation block experiment, we used 2 g of truffle inoculum per seedling.

### Seedling Harvests

Seedlings were harvested from the concentration experiment from all three sites in November 2015 (year 2) and from the Tift site only in February 2018 (year 5). Seedlings were harvested from the fumigation and inoculation block experiment only from the Tift site in November 2015 (year 2), October 2017 (year 4), and February 2018 (year 5). For each experiment, we arbitrarily harvested five healthy seedlings that were spread across each treatment area. Seedlings were selected because they were representative in size for each treatment and were dispersed across a treatment at each site, for each treatment, and at each sampling time for mycorrhizal analysis. Seedlings were carefully excavated with shovels in order to maximize the availability of fine ECM root tips. In years 4 and 5, we also used heavy machinery to remove seedlings due to the large size of their root systems. Harvested seedlings were placed in plastic bags on ice in the field for transport prior to storage at 3°C for no more than 5 days prior to processing. Seedling root systems were thoroughly washed with running tap water to maximize removal of soil and debris. Seedling fine roots were visually inspected to confirm that healthy ECM root tips were present and they were briefly scanned to qualitatively assess the diversity of different ECM morphotypes. When possible we noted the presence of ECM roots putatively colonized by *Tuber* species (smooth brown morphotypes with limited extrametrical hyphae) (Bonito et al., 2011). Following visual inspection, we sampled visible ECM fine-root tips of all morphotypes and their immediate subtending roots from each seedling. Specifically, all ECM fine-roots were sampled from young seedlings (year 2) whereas a subset of the root system was sampled from the 4 and 5 year old seedlings. The subset of the roots sampled in years 4 and 5 were the roots closest to the tree; roots >1 m away were severed and could not be excavated. Therefore, the roots we recovered were a subsample of the whole root system in these older trees. A maximum volume of 50 cm^3^ of roots were removed and stored in sterile 50 ml tubes at −80°C.

The volume of ECM root tips harvested in years 4 and 5 was smaller than in year 2 due to the loss of fine root tips that occurred during the removal of seedlings with larger root systems. Samples were lyophilized prior to DNA extraction. In preparation for lyophilization, the opening of each 50 mL tube was covered with a clean Kimwipe (Kimberly-Clark Worldwide, Inc., Irving, TX, United States) that was affixed with a rubber band to prevent sample loss when the vacuum was engaged. Ectomycorrhizal fine root tips were lyophilized for approximately 48 h with a Labconco FreeZone 2.5 Plus lyophilizer (Labconco Corp., Kansas City, MO, United States). After lyophilizing, seven autoclaved glass beads (3 mm diameter) were placed inside each 50 ml tube and the dried ECM fine roots were pulverized for 30 s at 1,500 rpm with a 1600 MiniG Bead Beater (SPEX SamplePrep, LLC, Metuchen, NH, United States). Once the ECM fine root tips were pulverized, the larger pieces of second order roots were removed using flame-sterilized forceps. Approximately 250 mg of the resulting root powder was transferred to tubes from the MoBio Soil Extraction kit (Qiagen, Venlo, Netherlands) for DNA extraction following the manufacturer’s recommendations. In order to improve DNA purity and conserve resources compared to commercial kits, samples from years 4 and 5 used the same input of lyophilized and pulverized ECM fine roots but employed a modified CTAB DNA extraction protocol with an initial phenol-chloroform step. In this modified protocol, the samples were left to incubate at room temperature in the phenol solution for 24 h before continuing the extraction according to the methods of Gardes and Bruns (1993).

### Fungal Community High Throughput Amplicon Sequencing (HTAS) and Bioinformatics

The resulting DNA was amplified with the fungal-specific polymerase chain reactions (PCR) primer ITS1F (Gardes and Bruns, 1993) and ITS2 (White et al., 1990); both primers were modified with Illumina Nextera v2 adapters (Illumina Inc., San Diego, CA, United States). We used a dual-index Illumina (Illumina, San Diego, CA, United States) protocol using Illumina Nextera v2 indices. PCRs were conducted in a total volume of 25 μL containing 0.5 μl Phusion High-Fidelity DNA Polymerase (Thermo Fisher Scientific, Waltham, MA, United States), 5 μl of 5× Phusion HF Buffer, 0.5 μL of 10 mM dNTPs, 1.25 μl of 10 μM each of the two primers, 1 μL of molecular grade 2% BSA (New England Biolabs, Ipswich, MA, United States), 14.5 μl of molecular grade water and 1 μl of the DNA template. The PCR negative control used DNA-sterile molecular grade water as the template. The PCR cycling conditions were: initial denaturation at 95°C for 1 min, followed by 35 cycles of denaturation at 95°C for 30 s, annealing at 54°C for 45 s, with a −0.1°C/cycle drop, and extension at 72°C for 1 min, with a final extension at 72°C for 7 min. PCR products were visualized through gel electrophoresis with a 2% agarose gel. Samples were equilibrated for molarity with a SequalPrep Normalization Plate Kit (Thermo Fisher, Waltham, MA, United States) for years 2 and 4. In year 5, products were cleaned with Select-a-Size Clean & Concentrate kit (Zymo Research, Irvine, CA, United States) following manufacturer recommendations for expected product size. Sample molarity was then assessed using a Qubit 4 Fluorometer with the Qubit dsDNA Broad Range Assay Kit (Thermo Fisher Scientific, Waltham, MA, United States). All samples were then mixed at an equimolar ratio. As a positive sequencing control, an in-house biological ITS mock community was included with samples for year 2, and a non-biological synthetic, single-copy ITS mock community (SYNMO; Palmer et al., 2018) was used in years 4 and 5. Samples were sequenced on three different runs with the Illumina MiSeq (Illumina, San Diego, CA, United States) device, using version 3 chemistry and the dual-indexed 2 × 300 bp setting at the University of Florida Interdisciplinary Center for Biotechnology Research^2^.

Sequences from all three Illumina sequencing runs were analyzed together with AMPtk v1.2.4 (Palmer et al., 2018). We pre-processed our merged, dual-indexed 2 × 300 MiSeq reads with USEARCH (version 9.2.64; Edgar, 2010) and then removed the forward and reverse ITS primers. We discarded any reads shorter than 150 bp, any reads longer than 300 bp were trimmed to 300 bp, and any reads shorter than 300 bp were padded with N’s from the 3′ end to help improve the clustering step (Palmer et al., 2018). Samples with fewer than 2,000 reads were dropped before clustering to avoid clustering errors (*n* = 4). Sequence reads were then quality-filtered with expected errors less than 1.0 (Edgar and Flyvbjerg, 2015), de-replicated, and clustered at 97% similarity to generate operational taxonomic units (OTUs) using UPARSE (Edgar, 2013). Following clustering, any padded N’s were removed, and the processed sequences were mapped to the OTUs. For samples of years 4 and 5, we used SYNMO (Palmer et al., 2018), a 12-member ITS single copy mock community composed of completely non-biological (i.e., synthetic) ITS sequences to account for observed rates of index bleed using the filter module in AMPtk. Because we did not use SYNMO for year 2 samples, we estimated index-bleed at 0.5% to be conservative. After index-bleed filtering, the OTUs were assigned taxonomy using the hybrid taxonomy algorithm in AMPtk. FUNGUILD (Nguyen et al., 2016), and its curated database of fungal OTU trophic modes, was used within the AMPtk pipeline to make preliminary designations of trophic mode for all OTUs. For the ECM fungi only dataset, we removed non-ECM fungi as well as OTUs that could only be determined to the family or order level when those taxonomic groups are not entirely composed of ECM fungi. Specifically, taxa of Pyronemataceae and Pezizaceae that could not be definitively identified as ECM or non-ECM were removed from the dataset according to Tedersoo and Smith (2013). Additionally, quality control steps were taken to apply a subtraction filter (−100) to all samples and all OTUs based on reads recovered from our positive and negative controls. Our ECM fungi only data matrix was also filtered to include only OTUs that occurred in more than one sample to reduce statistical noise from spurious OTUs of questionable trophic mode. Raw sequence data for samples used in this study are publicly available in the NCBI Sequence Read Archive (BioProject PRJNA714616).

### Statistical Analysis

We tested our experiments and their variables as follows: concentration of inoculum was tested by site and year, and the fumigation and inoculation block experiment tested by treatment, interactions between treatments, and the effect of year on community composition.

All analyses were performed with R Version 3.5.2 (R Core Team, 2017) using our presence/absence OTU matrix and select functions within the Vegan package (Oksanen et al., 2019) of R. We used the Raup-Crick distances calculated with function raupcrick (Chase et al., 2011) for “all fungi” and “ECM only fungi” multivariate analyses and ordinations. We also utilized the Shannon diversity calculation feature within the Vegan package to determine how fungal diversity changed over time. We examined diversity, using the Shannon diversity calculations, for the “all fungi” and “ECM only fungi” datasets independently at each year within the concentration rate and fumigation and inoculation block experiments at Tift as this was an experimental orchard.

To visualize our data, we performed non-metric multidimensional scaling (NMDS) with the metaMDS function. Permutational multiple analysis of variance tests (PERMANOVA) were performed using the adonis function (Anderson, 2001) to determine the statistical significance of the different variables of interest (fumigation, inoculation, concentration rate of truffle inoculum, and fumigation × inoculation) in the experiments with significance determined at the 0.05 level. Because PERMANOVA is sensitive to both centroid location and dispersion, we also analyzed multivariate homogeneity of group dispersions (Anderson, 2006) using the betadisper function.

## Results

### Soil Characteristics

Soil characteristics from the three sites at year 2 (2015) differed most in pH (5.99–6.5) and potassium levels (10.69–45.06) (Table 1).

### Fungal Communities Based on DNA Sequencing

We recovered sequences from 137 of our 170 samples (80.5%). For all fungi (including all sites, years and experiments) we recovered a total of 10,070,714 reads (mean 76,876 reads per pecan seedling sample, median 72,134 reads per sample, range 985–255,314 reads per samples) from our Illumina MiSeq runs after data filtering. Four samples were discarded from the dataset because they had fewer than 2,000 reads. This resulted in 1,899 fungal OTUs (mean 72.8 OTUs per sample, median 61 OTUs per sample, range 6–249 OTUs per sample) from the remaining 133 samples. Seven samples had no OTUs that were classified as ECM fungi so these samples were not included in the ECM only fungi analysis. For known ECM fungal communities, we recovered 6,604,367 reads, representing 65.6% of the total recovered reads (mean 53,261 reads per sample, median 46,866 reads per sample, range 394–218,151 reads per sample). We identified a total of 47 ECM fungi OTUs of the 1,322 total fungi OTUs (Supplementary Table 2) (mean 3.5 ECM OTUs per sample, median 3 ECM OTUs per sample, range 1–11 ECM OTUs per sample). The most commonly recovered taxa were: *T. lyonii* (Tuberaceae, Ascomycota), *Scleroderma* spp. (Sclerodermataceae, Basidiomycota), *Astraeus pteridis* (Diplocystaceae, Basidiomycota), and *Sphaerosporella* sp. (Pyronemataceae, Ascomycota). We also detected other Basidiomycota species within the Amanitaceae, Boletaceae, Gyroporaceae, Hydnangiaceae, Pisolithaceae, Thelephoraceae, and Ascomycota species within the Pezizaceae, Pyronemataceae, and Tuberaceae (Supplementary Table 2).

### Influence of Time Since Inoculation on Fungal Community Composition

To investigate the influence of time since inoculation on the fungal community and to examine potential successional changes, we tested whether time since inoculation had a statistically significant impact on the entire fungal community or for the ECM only community for both experiments that were conducted at Tift. For the concentration experiment, samples from different years had differences in community composition and multivariate dispersion for the entire fungal community (adonis: *p* < 0.0001, *pseudo-F* = 112.36, *r*^2^ = 0.5367; betadisper: *p* < 0.0001, *F* = 83.115) and also for the ECM fungi only dataset (adonis: *p* < 0.0001, *pseudo-F* = 34.932, *r*^2^ = 0.31209; betadisper: *p* < 0.0001, *F* = 16.031). We also found significant differences in community composition and multivariate dispersion for the all fungi communities in the fumigation and inoculation block experiment (adonis: *p* < 0.0001, *pseudo-F* = 27.288, *r*^2^ = 0.3276; betadisper: *p* < 0.0001, *F* = 46.944). We detected significant differences between the community composition of the ECM only fungi in different years (adonis: *p* = 0.0401, *pseudo-F* = 2.8039, *r*^2^ = 0.0575) but there were not significant differences in the multivariate dispersion.

### Site Influences on Fungal Community Composition

Because we collected samples from three different sites for our concentration experiment in year 2, we tested whether site significantly influenced either the entire fungal community or the ECM fungi community. Site had a statistically significant impact on community composition and multivariate dispersion on both the entire fungal community (adonis: *p* < 0.0001, *pseudo-F* = 94.1220, *r*^2^ = 0.7771; betadisper: *p* = 0.0159, *F* = 4.3963) and the ECM only fungal community (adonis: *p* < 0.0001, *pseudo-F* = 7.4179, *r*^2^ = 0.2037; betadisper: *p* = 0.0123, *F* = 4.7668).

### Effects of Inoculum Concentration on the Root Fungal Community of Pecan Seedlings

Inoculum concentration had a statistically significant impact on community composition and multivariate dispersion on the entire fungal community and also significant effects on multivariate dispersion at Tift in year 2 (adonis: *p* = 0.0105, *pseudo-F* = 3.9190, *r*^2^ = 0.5833; betadisper: *p* = 0.0002, *F* = 11.4170), Berrien in year 2 (betadisper: *p* = 0.0299, *F* = 3.1025), Screven in year 2 (adonis: *p* < 0.0001, *pseudo-F* = 16.7170, *r*^2^ = 0.8069; betadisper: *p* = 0.0349, *F* = 3.0075), and Tift in year 5 (adonis: *p* = 0.0002, *pseudo-F* = 11.8640, *r*^2^ = 0.7479, betadisper: *p* = 0.0005, *F* = 7.3783) (Table 2).

**TABLE 2:** Ecological statistics, sample size, and number of operational taxonomic units (OTUs) for all sites and all years (year 2 = 2015, year 5 = 2018) for the concentration rate experiment.

**Year**	**Site**	**Community**	**Variable**	**Adonis *r*^2^**	**Adonis *Pseudo-F***	**Adonis *p*-value**	**Betadisper *F*-value**	**Betadisper *p*-value**	***n*=**	**# OTUs**
2	Tift	All Fungi	Concentration	0.5833	3.9190	**0.0105**	11.4170	**0.0002**	20	661
		Only ECM		0.4172	1.9683	0.0619	2.1257	0.1597	16	19
2	Berrien	All Fungi	Concentration	0.4078	2.8926	0.0665	3.1025	**0.0299**	27	631
		Only ECM		0.2922	1.7337	**0.0398**	1.7070	0.1923	27	12
2	Screven	All Fungi	Concentration	0.8069	16.7170	** < 0.0001**	3.0075	**0.0349**	26	572
		Only ECM		0.7336	11.0140	** < 0.0001**	0.2887	0.8812	26	17
5	Tift	All Fungi	Concentration	0.7479	11.8640	**0.0002**	7.3783	**0.0005**	26	495
		Only ECM		0.6251	6.0015	** < 0.0001**	13.1490	** < 0.0001**	24	25

*For each site and each year we analyzed two different communities, one that included all fungi together (“all fungi”) and a subset of the community that included only ectomycorrhizal (ECM) fungi (“only ECM”). Fungal communities were analyzed based on Raup-Crick distances. Values in bold are statistically significant.*

Inoculation concentration had a statistically significant effect on the ECM only fungal community composition at Berrien in year 2 (adonis: *p* = 0.0398, *pseudo-F* = 1.7337, *r*^2^ = 0.2922), and Screven in year 2 (adonis: *p* < 0.0001, *pseudo-F* = 11.0140, *r*^2^ = 0.7336), with no effects of multivariate dispersion. For Tift at year 5, inoculum concentration had a statistically significant impact on community composition and multivariate dispersion on the ECM only fungal community (adonis: *p* < 0.0001, *pseudo-F* = 6.0015, *r*^2^ = 0.6251; betadisper: *p* < 0.0001, *F* = 13.1490) (Table 2).

**FIGURE 1:**
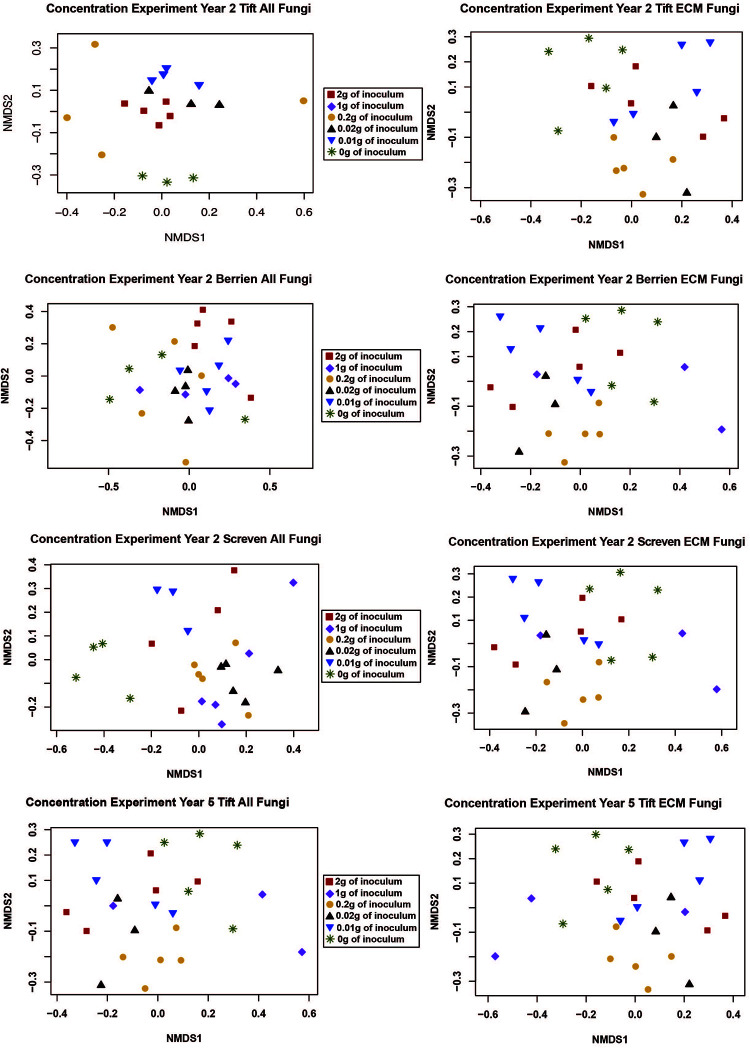
NMDS ordination for the concentration experiment with both the “all fungi” and “ECM fungi only” datasets using the Raup-Crick distance metric, stress <0.2, and 2–3 dimensions. The only sample for the 1 g treatment at Tift was removed from the ordination because it was an extreme outlier.

We performed a NMDS ordination to visualize how inoculum concentration impacted the composition of the ECM and all fungal communities. However, no clear patterns emerged (Figure 1).

### Effects of Fumigation, Inoculation, and the Interaction Between Fumigation and Inoculation on the Fungal Communities on the Roots of Pecan Seedlings

Fumigation had a statistically significant impact on community composition and multivariate dispersion during all 3 years (years 2, 4, and 5) for the entire fungal community (Table 3; adonis: *p* = 0.0009, <0.0001, 0.0069, *pseudo-F* = 13.317, 47.991, 6.6814, *r*^2^ = 0.5476, 0.7384, 0.2946; betadisper: *p* = 0.0001, 0.0001, 0.0001, *F* = 21.6690, 22.9290, 21.0700) and the ECM only fungal community and resulted in significantly different ECM only fungal community composition in different treatments (Table 3; adonis: *p* = 0.0006, 0.0008, 0.0020, *pseudo-F* = 7.0891, 8.6354, 5.4107, *r*^2^ = 0.3919, 0.3654, 0.2527) with no effects of multivariate dispersion (Table 3). Inoculation did not have a statistically significant effect on the entire fungal community composition during any of the 3 years but we did detect statistically significant differences in multivariate dispersion (betadisper: *p* = <0.0001, <0.0001, <0.0001, *F* = 28.5870, 29.5100, 29.0950). For the ECM fungi only dataset, inoculation had a statistically significant impact on ECM fungal community composition in year 2 only (Table 3; adonis: *p* = 0.0188, *pseudo-F* = 3.7246, *r*^2^ = 0.2530) but not in years 4 or 5.

**TABLE 3:** Ecological statistics, sample size, and number of operational taxonomic units (OTUs) for all years (year 2 = 2015, year 4 = 2017, year 5 = 2018) of the fumigation and inoculation experiment that was conducted at the Tift experimental orchard in Tift County, Georgia, United States.

**Year**	**Community**	**Variable**	**Adonis *r*^2^**	**Adonis *Pseudo-F***	**Adonis *p*-value**	**Betadisper *F*-value**	**Betadisper *p*-value**	***n*=**	**# OTUs**
2	All Fungi	Fumigation	0.5476	13.3170	**0.0009**	21.6690	**0.0001**	13	484
		Inoculation	0.1688	2.2341	0.1602	28.5870	** < 0.0001**		
		F × I	0.2208	10942	** < 0.0001**	N/A	N/A		
	Only ECM	Fumigation	0.3919	7.0891	**0.0006**	0.2644	0.6173	13	11
		Inoculation	0.2530	3.7246	**0.0188**	0.2756	0.6100		
		F × I	0.1696	7.1019	** < 0.0001**	N/A	N/A		
4	All Fungi	Fumigation	0.7384	47.9910	** < 0.0001**	22.9290	0.0001	19	632
		Inoculation	0.1311	2.5653	0.1595	29.5100	** < 0.0001**		
		F × I	0.0657	67.4700	** < 0.0001**	N/A	N/A		
	Only ECM	Fumigation	0.3654	8.6354	**0.0008**	0.0058	0.9403	17	16
		Inoculation	0.1039	1.7384	0.1627	0.0927	0.7649		
		F × I	0.0310	0.7907	0.5281	N/A	N/A		
5	All Fungi	Fumigation	0.2946	6.6814	**0.0069**	21.0700	0.0001	18	400
		Inoculation	0.1126	2.0311	0.2381	29.0950	** < 0.0001**		
		F × I	0.1391	4.2170	0.0425	N/A	N/A		
	Only ECM	Fumigation	0.2527	5.4107	**0.0020**	0.9793	0.3371	18	27
		Inoculation	0.0395	0.6576	0.6047	0.0107	0.9187		
		F × I	0.1060	2.4784	0.0822	N/A	N/A		

*For each site and each year we analyzed two different communities, one that included all fungi together (“all fungi”) and a subset of the community that included only ectomycorrhizal (ECM) fungi (“only ECM”). Fungal communities were analyzed based on Raup-Crick distances. Values in bold are statistically significant.*

The interaction between fumigation and inoculation had a statistically significant impact on the entire fungal community composition in all 3 years; in year 2 (adonis: *p* = <0.0001, *pseudo-F* = 1094, *r*^2^ = 0.2208), in year 4 (adonis: *p* = <0.0001, *pseudo-F* = 67.4700, *r*^2^ = 0.0657), and in year 5 (adonis: *p* = 0.0425, *pseudo-F* = 4.2170, *r*^2^ = 0.1391) (Table 3). Due to the limitations of the betadisper function, we could not test for multivariate dispersion on the interaction terms.

**FIGURE 2:**
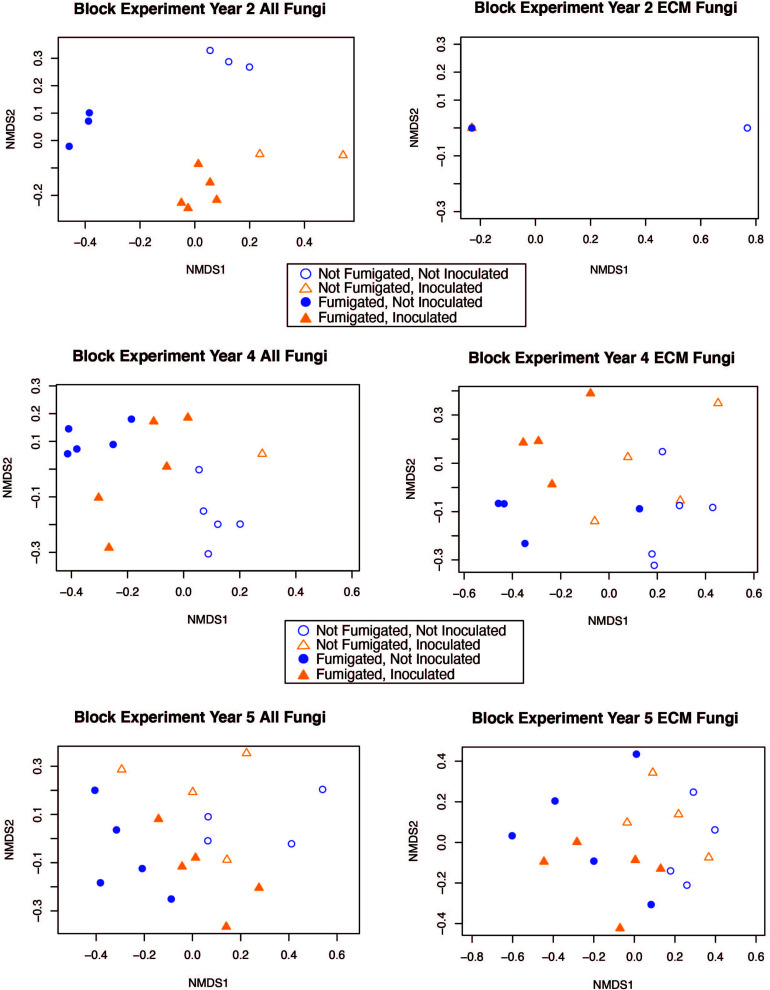
NMDS ordination for the fumigation and inoculation block experiment with both the “all fungi” and “ECM fungi only” datasets using the Raup-Crick distance metric, stress <0.2, and 2–3 dimensions.

The interaction between fumigation and inoculation had a statistically significant impact on the ECM fungal community composition only in year 2 (adonis: *p* = <0.0001, *pseudo-F* = 7.1019, *r*^2^ = 0.1696) (Table 3). In order to visualize the relationships among samples from the fumigation and inoculation block experiment, we performed an NMDS ordination and observed clear separation of samples based on the four different treatments in year 2 for the entire fungal community. However, the communities generally converged in years 4 and 5 for both the all fungi community and the ECM fungi only community (Figure 2).

### Effects of Inoculation Concentration on the Presence of *Tuber lyonii* on the Roots of Pecan Seedlings

Visual examination of pecan seedling fine roots showed ample ECM fungi colonization and confirmed the presence of smooth brown morphotypes consistent with colonization by a *Tuber* species. To quantify colonization by *T. lyonii* we used the sequence read abundance from Illumina sequencing as follows. We calculated the percentage of *T. lyonii* sequence reads in each sample as *T. lyonii* reads per sample/total number of reads per sample × 100. For any given treatment we then used the median of the percentage of *T. lyonii* sequences as an indicator of whether or not *T. lyonii* became well established as an ECM partner on pecan seedlings in this treatment. The median of the percentage of *T. lyonii* sequences was used rather than the mean of the percentage of *T. lyonii* reads because the median results were less prone to the effect of outliers from small sample sizes and had less variation in the number of *T. lyonii* reads per sample. Median percentage of *T. lyonii* reads ranged from <1% (0.2 g of inoculum at Tift in year 2) to 92% (1 g of inoculum at Berrien in year 2), demonstrating a wide variation in the relative number of *T. lyonii* reads compared to the total number of reads per treatment (Table 4). For Tift in year 4, the percentage of median reads of *T. lyonii* ranged from 19% (1 g) to none (0.1 and 0.01 g) (Table 4).

**TABLE 4:** Year, site, treatment, sample size, median percent of *T. lyonii* reads per treatment, and median total reads per treatment for all fungi from the concentration rate experiment (year 2 = 2015, year 5 = 2018).

**Year**	**Site**	**Concentration (grams perseedling)**	**Sample size (number of seedlings)**	**Median percent of reads of *T. lyonii* Per Treatment**	**Median total reads per treatment**
2	Tift	2	5	9%	103,373
		1	0	N/A	47,413
		0.2	3	<1%	63,159
		0.1	3	2%	80,697
		0.01	2	1%	62,916
2	Berrien	2	5	87%	85,745
		1	4	92%	117,292
		0.2	5	47%	110,146
		0.1	4	65%	138,947
		0.01	5	74%	64,934
2	Screven	2	4	61%	104,235
		1	5	69%	146,074
		0.2	4	63%	83,177
		0.1	5	61%	87,295
		0.01	3	12%	112,103
5	Tift	2	5	3%	73,192
		1	3	19%	41,680
		0.2	5	N/A	72,444
		0.1	3	N/A	77,242
		0.01	4	N/A	54,716

### Effects of Inoculation and Fumigation on the Presence of *Tuber lyonii* on Roots of Pecan Seedlings

To look at the influence of our fumigation and inoculation treatments on the presence of *T. lyonii*, we also calculated the median percentage of *T. lyonii* sequences for the fumigation and inoculation block experiment. The median percentage of *T. lyonii* reads with fumigated and inoculated seedlings ranged from 3% (year 5) to 43% (year 4) demonstrating a wide variation in the relative number of reads from *T. lyonii* (Table 5). The median percentage of *T. lyonii* reads with non-fumigated but inoculated seedlings ranged from 16% (year 4) to 0 (year 5) (Table 5).

**TABLE 5:** Year, site, treatment, sample size, median percent of *T. lyonii* reads per treatment, and median total reads per treatment for all fungi from the fumigation and inoculation experiment (year 2 = 2015, year 4 = 2017, year 5 = 2018).

**Year**	**Site**	**Treatment**	**Sample size (Number of seedlings)**	**Median percent of reads of *T. lyonii* Per Treatment**	**Median total reads per treatment**
2	Tift	F+I+	5	9%	103,373
		F−I+	2	6%	46,867
		F+I−	3	N/A	68,214
		F−I−	2	N/A	59,177
4	Tift	F+I+	5	43%	30,878
		F−I+	4	16%	12,242
		F+I−	5	N/A	31,973
		F−I−	5	N/A	24,924
5	Tift	F+I+	5	3%	73,192
		F−I+	4	0	67,162
		F+I−	5	N/A	80,100
		F−I−	4	N/A	62,832

## Discussion

**FIGURE 3:**
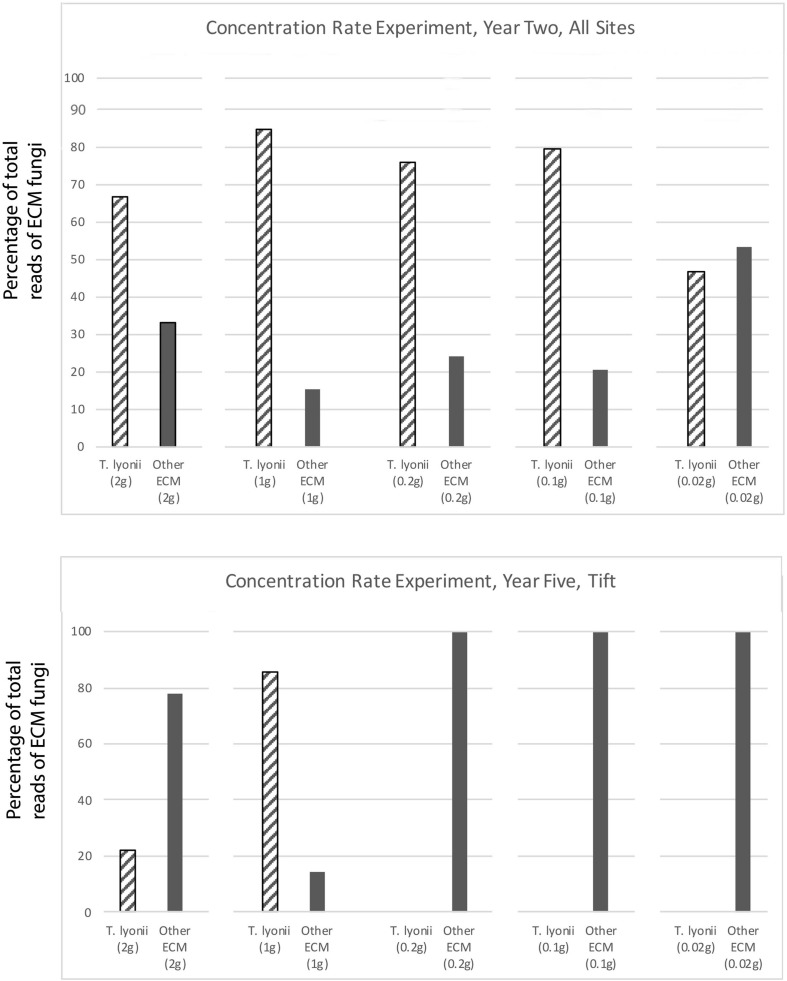
Results of our concentration experiment for year 2 at all sites and for year 5 at the Tift site. This figure depicts the percentage of total sequence reads that were from *T. lyonii* versus all other ECM fungi in each of the different *T. lyonii* inoculum treatment levels (2, 1, 0.2, 0.1, and 0.02 g).

**FIGURE 4:**
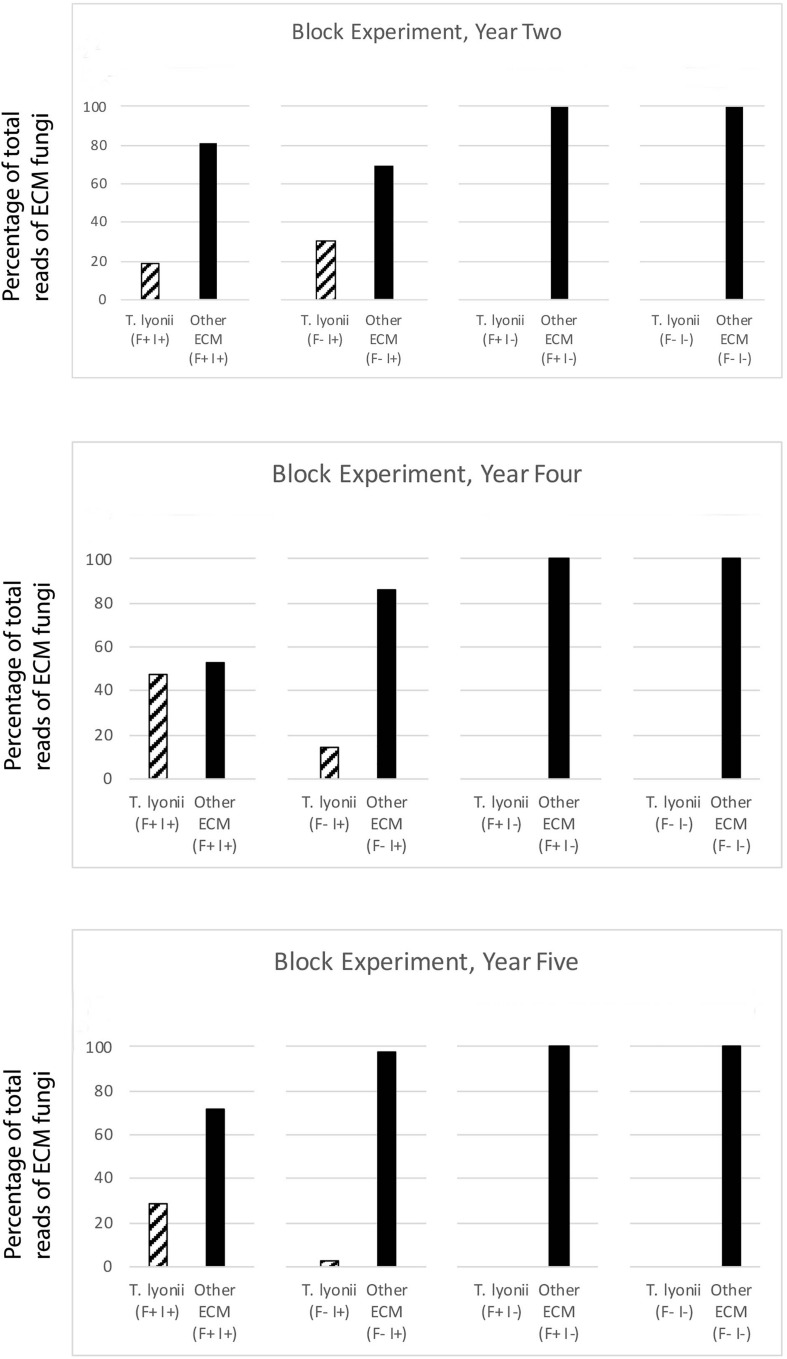
Results of the fumigation and inoculation block experiment at Tift for year 2, 4, and 5. This figure depicts the percentage of total sequence reads that were from *T. lyonii* versus all other ECM fungi in each of the different treatments: fumigation and inoculation (F+, I+), fumigation and no inoculation (F+, I–), no fumigation and inoculation (F–, I+), and no fumigation and no inoculation (F–, I–).

The results of our 5-year experiments demonstrate that it is feasible to inoculate pecan seedlings with the pecan truffle (*T. lyonii*) in the field under certain management and environmental conditions. We regularly detected DNA of *T. lyonii* in inoculated seedlings, and this truffle remained a part of the fungal community throughout the 5 years of the experiment after inoculation of seeds with 1–2 g of *T. lyonii* spores (Tables 4, 5 and Figures 3, 4). Our careful root washing followed by visualization of healthy ECM root colonization confirms that the *T. lyonii* sequences from the sampled pecan seedling root systems represent fresh and viable ECM root tips rather than hyphae or spores in the surrounding soil. This is the first study to directly inoculate *Tuber* species onto seedlings in commercial pecan nurseries without first utilizing a greenhouse or other controlled system prior to out-planting inoculated seedlings. Taken overall, our results suggest that 1 g of truffle inoculum or more should be sufficient to initiate colonization and persistence on ECM root tips by *T. lyonii* (Figures 3, 4).

We found that pecan seedlings inoculated with a wide density of truffle spore inoculum became ectomycorrhizal with *T. lyonii* in the field based on our high throughput amplicon sequencing (HTAS) approach. In fact, during year 2 of the experiment *T. lyonii* was the most abundant fungus on roots of pecan seedlings that were planted in fumigated soil, even when as little as 0.1 g of truffle inoculum was used (Figures 3, 4). However, our results from year 5 suggests *T. lyonii* declined based on read abundance and it was no longer the most abundant fungus on the roots of pecan seedlings at Tift, except for our 1 g inoculation treatment in the concentration experiment (Figures 3, 4). The number of *T. lyonii* reads in year 2 was mostly correlated with the inoculum concentration (Table 4), suggesting that more inoculum generally produces higher colonization rates. However, in year 5 the high rate of *T. lyonii* colonization in the 1 g per seedling treatment (versus the higher inoculation rate of 2 g per seedling) is potentially due to sampling issues at Tift that resulted in one sample having no *T. lyonii* reads and three other samples having relatively low read counts for *T. lyonii* (median <12% of reads). These results may represent true decline in *T. lyonii* abundance but might also be attributed to sampling issues. As the seedlings grew during the course of this experiment, it became increasingly more difficult to obtain representative ECM root tip samples. Two-year-old seedlings could be comprehensively sampled because they were small enough to extract almost the entire root system with a shovel. However, 5-year-old seedlings had extensive root systems that extended in a radius of greater than one meter and sometimes we observed roots more than two meters deep. Heavy machinery was therefore required to remove 5-year-old trees from the ground, resulting in substantial losses of fine ECM root tips. Also, because the root systems were so large in 5-year-old plants, it was difficult to know what percentage of the ECM root tips were being sampled.

While the long-term effects of nursery field inoculation on seedlings that have been removed from the nursery and out-planted are not yet known, we are confident that *T. lyonii*-colonized roots were present at year 2, when seedlings would typically be transplanted into production orchards. Our data also suggest that seedlings should be transplanted after 2 years of growth in the nursery. Two-year-old seedlings have sufficiently small root systems to allow for efficient transplantation of ECM root tips that are colonized by *T. lyonii* whereas larger root systems are more likely to have been colonized by other ECM fungi and are also harder to transplant with their colonized root tips intact.

Our experiments focused on inoculating pecan seedlings with the pecan truffle in the nursery environment, but our results also shed light on the successional changes in fungal communities associated with the roots of pecan seedlings over time. Interestingly, the number of fungal OTUs recovered from our concentration experiment was highest for the entire fungal community at year 2 (*n* = 661 OTUs) as compared to year 5 (*n* = 495 OTUs). This is presumably due to displacement of root endophytes, saprobes and other rhizosphere fungi as ECM fungal biomass increased with the increased size and vigor of host seedlings. As expected, however, the number of ECM fungi OTUs increased over the course of our experiment with only 19 total ECM OTUs detected in year 2 seedlings versus 25 ECM OTUs in year 5 seedlings (Table 2). While we observed changes in the number of OTUs recovered, the differences in community composition were not statistically significant when evaluated with Shannon diversity indices for any experiment, guild, or year (Supplementary Table 3 and Supplementary Figure 1). Throughout the experiment the main competitors of *T. lyonii* were primarily disturbance-adapted ECM fungi in the Boletales (*Scleroderma* spp., *A. pteridis*, and *Pisolithus*) and *Sphaerosporella* (Pezizales) (Supplementary Tables 1, 2). At the same time, we also observed a decrease in the median percentage of *T. lyonii* reads per sample over time (Tables 4, 5). Together these results suggest that there are successional changes in the fungal communities on pecan seedlings over time in the nursery environment, with *T. lyonii* generally becoming less dominant (Table 4). As pecan seedlings make new root tips, it appears that newly arriving ECM fungi are likely to compete directly with *T. lyonii* for both roots and space in the rhizosphere, as has been shown with other ECM fungi in other systems (Kennedy et al., 2011; Bonito et al., 2012; Pickles et al., 2012; Smith et al., 2018). These competitor ECM fungi may or may not present a threat to future proliferation and fruiting of *T. lyonii*. It is notable that species of *Scleroderma* and *Pisolithus* are among the most common ECM fungi found fruiting in pecan orchards and are also regularly detected on the ECM roots of mature pecan trees (Ge et al., 2017, M. Smith personal communication, Supplementary Table 2). Future studies are clearly needed to see if *T. lyonii* persists along with competitor ECM fungi and whether agricultural management practices, such as increasing the soil pH, removal of understory vegetation and regular soil disturbance, might favor proliferation and persistence of *T. lyonii* over other ECM fungi.

Fungal communities from each of the different treatments in the fumigation and inoculation block experiment were strongly clustered in year 2 because the treatments had a strong impact on fungal community composition. However, this pattern became less distinct during year 4 and 5 as fungal communities converged and more OTUs were detected in ECM fungal communities (although Shannon diversity indices were not statistically different over time) (Table 2, Supplementary Table 3, and Supplementary Figure 1). Although fungal community composition in different treatments generally converged over time, the fumigated and non-fumigated samples remained clearly differentiated from each other throughout the 5-year duration of the experiment (Figure 2). This suggests that the fumigation treatment using a potent, full-spectrum biocide has legacy effects which last over the course of 5 years and perhaps even longer. Given the impact of fumigation on community composition within treatments, it would be interesting to see how long the effects of the fumigation treatment could be observed on the fungal community composition.

Inferring relative abundance of taxa based on sequence abundance is fraught with challenges (Palmer et al., 2018; Jusino et al., 2019). Although studies have demonstrated that read abundance from HTAS platforms is not necessarily indicative of species abundance, there is evidence that HTAS sequence abundance can be interpreted as “semi-quantitative” (Amend et al., 2010). While we relied on abundance in this case due to small sample sizes, the data from presence/absence of *T. lyonii* also corroborates our findings and results. In the future, studies with more seedlings that can rely on presence/absence or on biomass estimates that count colonized root tips will be important to verify our results.

While our study focused on the potential for commercial pecan and pecan truffle orchards, these data and techniques may apply to other *Tuber* species. The capacity to successfully inoculate ECM fungi of commercial interest *in situ* and cultivate these fungi in agricultural landscapes, could potentially encourage the reforestation of barren or non-arable lands that could not only help to restore forest environments, but also provide an added economic benefit to growers and land owners.

## Data Availability Statement

The data presented in the study are deposited in the NCBI Sequence Read Archive repository, https://www.ncbi.nlm.nih.gov/sra/, BioProject PRJNA714616.

## Author Contributions

TB, GB, and MS contributed to the concept and design of the experiment. TB and MS set up the experiment. AG, TB, MJ, and AM performed the research and data analysis. AG and MS drafted the first version of the manuscript. All authors contributed to editing and preparation of the final manuscript.

## Conflict of Interest

The authors declare that the research was conducted in the absence of any commercial or financial relationships that could be construed as a potential conflict of interest.

## Footnotes

1 http://sfyl.ifas.ufl.edu/agriculture/soil-testing/

2 http://www.biotech.ufl.edu/

## References

[B1] Adams J. C. Thielges B. A. (1978). *Seed Treatment for Optimum Pecan Germination.* Boise, ID: Tree Plant Notes US Forest Service.

[B2] Amend A. S. Seifert K. A. Bruns T. D. (2010). Quantifying microbial communities with 454 pyrosequencing: does read abundance count? *Mol. Ecol.* 19 5555–5565. 10.1111/j.1365-294x.2010.04898.x 21050295

[B3] Anderson M. J. (2001). A new method for non-parametric multivariate analysis of variance. *Aust. Ecol.* 26 32–46. 10.1111/j.1442-9993.2001.01070.pp.x

[B4] Anderson M. J. (2006). Distance-based tests for homogeneity of multivariate dispersions. *Biometrics* 62 245–253. 10.1111/j.1541-0420.2005.00440.x 16542252

[B5] Benucci G. M. Bonito G. Falini L. B. Bencivenga M. Donnini D. (2012). Mycorrhizal inoculation of pecan seedlings with some marketable truffles. *Acta Mycol.* 47 179–184. 10.5586/am.2012.022

[B6] Bonet J. A. Fischer C. R. Colinas C. (2006). Cultivation of black truffle to promote reforestation and land-use stability. *Agron. Sustain. Dev.* 26 69–76. 10.1051/agro:2005059

[B7] Bonet J. A. Oliach D. Fischer C. Olivera A. Martinez de Aragon J. Colinas C. (2009). Cultivation methods of the black truffle, the most profitable mediterranean non-wood forest product; a state of the art review. *EFI Proc.* 57 57–71.

[B8] Bonito G. Brenneman T. Vilgalys R. (2011). Ectomycorrhizal fungal diversity in orchards of cultivated pecan (*Carya illinoinensis*; Juglandaceae). *Mycorrhiza* 21 601–612. 10.1007/s00572-011-0368-0 21369784

[B9] Bonito G. Smith M. E. Brenneman T. Vilgalys R. (2012). Assessing ectomycorrhizal fungal spore banks of truffle producing soils with pecan seedling trap-plants. *Plant Soil* 356 357–366. 10.1007/s11104-012-1127-5

[B10] Bonito G. M. Gryganskyi A. P. Trappe J. M. Vilgalys R. (2010). A global meta-analysis of Tuber ITS rDNA sequences: species diversity, host associations and long-distance dispersal. *Mol. Ecol.* 19 4994–5008. 10.1111/j.1365-294x.2010.04855.x 21040049

[B11] Chase J. M. Kraft N. J. Smith K. G. Vellend M. Inouye B. D. (2011). Using null models to disentangle variation in community dissimilarity from variation in α-diversity. *Ecosphere* 2 1–11.

[B12] Claus R. Hoppen H. O. Karg H. (1981). The secret of truffles: a steroidal pheromone? *Cell. Mol. Life Sci.* 37 1178–1179. 10.1007/bf01989905

[B13] Edgar R. C. (2010). Search and clustering orders of magnitude faster than BLAST. *Bioinformatics* 26 2460–2461. 10.1093/bioinformatics/btq461 20709691

[B14] Edgar R. C. (2013). UPARSE: highly accurate OTU sequences from microbial amplicon reads. *Nat. Methods* 10:996. 10.1038/nmeth.2604 23955772

[B15] Edgar R. C. Flyvbjerg H. (2015). Error filtering, pair assembly and error correction for next-generation sequencing reads. *Bioinformatics* 31 3476–3482. 10.1093/bioinformatics/btv401 26139637

[B16] Elliott T. F. Jusino M. A. Trappe J. M. Lepp H. Ballard G. A. Bruhl J. J. (2019). A global review of the ecological significance of symbiotic associations between birds and fungi. *Fungal Divers.* 98 161–194. 10.1007/s13225-019-00436-3

[B17] Eure P. M. Culpepper A. S. (2017). Bell pepper and weed response to dimethyl disulfide plus chloropicrin and herbicide systems. *Weed Technol.* 31 694–700. 10.1017/wet.2017.74

[B18] Gardes M. Bruns T. D. (1993). ITS primers with enhanced specificity for basidiomycetes-application to the identification of mycorrhizae and rusts. *Mol. Ecol.* 2 113–118. 10.1111/j.1365-294x.1993.tb00005.x 8180733

[B19] Ge Z. W. Brenneman T. Bonito G. Smith M. E. (2017). Soil pH and mineral nutrients strongly influence truffles and other ectomycorrhizal fungi associated with commercial pecans (*Carya illinoinensis*). *Plant Soil* 418 493–505. 10.1007/s11104-017-3312-z

[B20] Hanlin R. T. Wu M. Brenneman T. B. (1989). The occurrence of Tuber texense in Georgia. *Mycotaxon* 34 387–394.

[B21] Jusino M. A. Banik M. T. Palmer J. M. Wray A. K. Xiao L. Pelton E. (2019). An improved method for utilizing high-throughput amplicon sequencing to determine the diets of insectivorous animals. *Mol. Ecol. Resour.* 19 176–190. 10.1111/1755-0998.12951 30281913

[B22] Kennedy P. G. Higgins L. M. Rogers R. H. Weber M. G. (2011). Colonization-competition tradeoffs as a mechanism driving successional dynamics in ectomycorrhizal fungal communities. *PLoS One* 6:e25126. 10.1371/journal.pone.0025126 21949867 PMC3176321

[B23] Luoma D. L. Trappe J. M. Claridge A. W. Jacobs K. M. Cazares E. F. (2003). “Relationships among fungi and small mammals in forested ecosystems,” in *Mammal Community Dynamics in Western Coniferous Forests: Management and Conservation*, ed. Zabel C. J. (Cambridge: Cambridge University Press), 343–373. 10.1017/cbo9780511615757.011

[B24] Marozzi G. Sánchez S. Benucci G. M. Bonito G. Falini L. B. Albertini E. (2017). Mycorrhization of pecan (*Carya illinoinensis*) with black truffles: *Tuber* melanosporum and *Tuber brumale*. *Mycorrhiza* 27 303–309. 10.1007/s00572-016-0743-y 27838857

[B25] Murat C. (2015). Forty years of inoculating seedlings with truffle fungi: past and future perspectives. *Mycorrhiza* 25 77–81. 10.1007/s00572-014-0593-4 24989673

[B26] Nguyen N. H. Song Z. Bates S. T. Branco S. Tedersoo L. Menke J. (2016). FUNGuild: an open annotation tool for parsing fungal community datasets by ecological guild. *Fungal Ecol.* 20 241–248. 10.1016/j.funeco.2015.06.006

[B27] Olivier J. M. Savignac J. C. Sourzat P. (2002). *Truffe et Trufficulture.* Périgueux: Fanlac.

[B28] Oksanen J. Blanchet F. G. Friendly M. Kindt R. Legendre P. McGlinn D. (2019). vegan: Community Ecology Package. *R package version 2.5-4.* Available online at: https://CRAN.R-project.org/package=vegan.

[B29] Palmer J. M. Jusino M. A. Banik M. T. Lindner D. L. (2018). Non-biological synthetic spike-in controls and the AMPtk software pipeline improve mycobiome data. *PeerJ* 6:e4925. 10.7717/peerj.4925 29868296 PMC5978393

[B30] Pickles B. J. Genney D. R. Anderson I. C. Alexander I. J. (2012). Spatial analysis of ectomycorrhizal fungi reveals that root tip communities are structured by competitive interactions. *Mol. Ecol.* 21 5110–5123. 10.1111/j.1365-294x.2012.05739.x 22971047

[B31] R Core Team (2017). *R: A Language and Environment for Statistical Computing.* Vienna: R Foundation for Statistical Computing.

[B32] Samils N. Olivera A. Danell E. Alexander S. J. Fischer C. Colinas C. (2008). The socioeconomic impact of truffle cultivation in rural Spain 1. *Econ. Bot.* 62 331–340. 10.1007/s12231-008-9030-y

[B33] Smith G. R. Steidinger B. S. Bruns T. D. Peay K. G. (2018). Competition–colonization tradeoffs structure fungal diversity. *ISME J.* 12 1758–1767. 10.1038/s41396-018-0086-0 29491493 PMC6018791

[B34] Smith S. E. Read D. J. (2008). *Mycorrhizal Symbiosis.* San Diego, CA: Elsevier.

[B35] Sourzat P. Muratet G. Schneider J. P. (1990). “Observations sur le statut mycorhizien de jeunes arbres truffiers dans un essai de désinfection du sol au bromure de méthyle,” in *Proceedings of the Atti del 2° Congresso Internazionale sul Tartufo*, Vol. 1988 (Spoleto), 283–288.

[B36] Splivallo R. Deveau A. Valdez N. Kirchhoff N. Frey-Klett P. Karlovsky P. (2015). Bacteria associated with truffle-fruiting bodies contribute to truffle aroma. *Environ. Microbiol.* 17 2647–2660. 10.1111/1462-2920.12521 24903279

[B37] Splivallo R. Ottonello S. Mello A. Karlovsky P. (2011). Truffle volatiles: from chemical ecology to aroma biosynthesis. *New Phytol.* 189 688–699. 10.1111/j.1469-8137.2010.03523.x 21287717

[B38] Tedersoo L. Smith M. E. (2013). Lineages of ectomycorrhizal fungi revisited: Foraging strategies and novel lineages revealed by sequences from belowground. *Fungal Biol. Rev.* 27 83–99. 10.1016/j.fbr.2013.09.001

[B39] Trappe J. M. Molina R. Luoma D. L. Cázares E. Pilz D. Smith J. E. (2009). *Diversity, Ecology, and Conservation of Truffle Fungi in Forests of the Pacific Northwest.* Portland, OR: United States Department of Agriculture.

[B40] Vašutová M. Mleczko P. López-García A. Maček I. Boros G. Ševčík J. (2019). Taxi drivers: the role of animals in transporting mycorrhizal fungi. *Mycorrhiza* 29 413–434. 10.1007/s00572-019-00906-1 31292712

[B41] Wells L. (2018). *UGA Pecan Extension | Preliminary Acreage and Crop Loss Values for Georgia Pecans After Hurricane Michael.* Available online at: https://site.extension.uga.edu/pecan/2018/10/preliminary-acreage-and-crop-loss-values-for-georgia-pecans-after-hurricane-michael/ (accessed Jun 8, 2019).

[B42] White T. J. Bruns T. Lee S. J. W. T. Taylor J. L. (1990). Amplification and direct sequencing of fungal ribosomal RNA genes for phylogenetics. *PCR Protoc.* 18 315–322. 10.1016/b978-0-12-372180-8.50042-1

