# Supplementary material for: Effects of Field Fumigation and Inoculation With the Pecan Truffle (Tuber lyonii) on the Fungal Community of Pecan (Carya illinoinensis) Seedlings Over 5 Years
Source: Front Microbiol. 2021 May 13;12:661515. doi: 10.3389/fmicb.2021.661515 (PMC8155716; doi:10.3389/fmicb.2021.661515)
Supplement: Supplementary file 1 [file Data_Sheet_1.PDF]

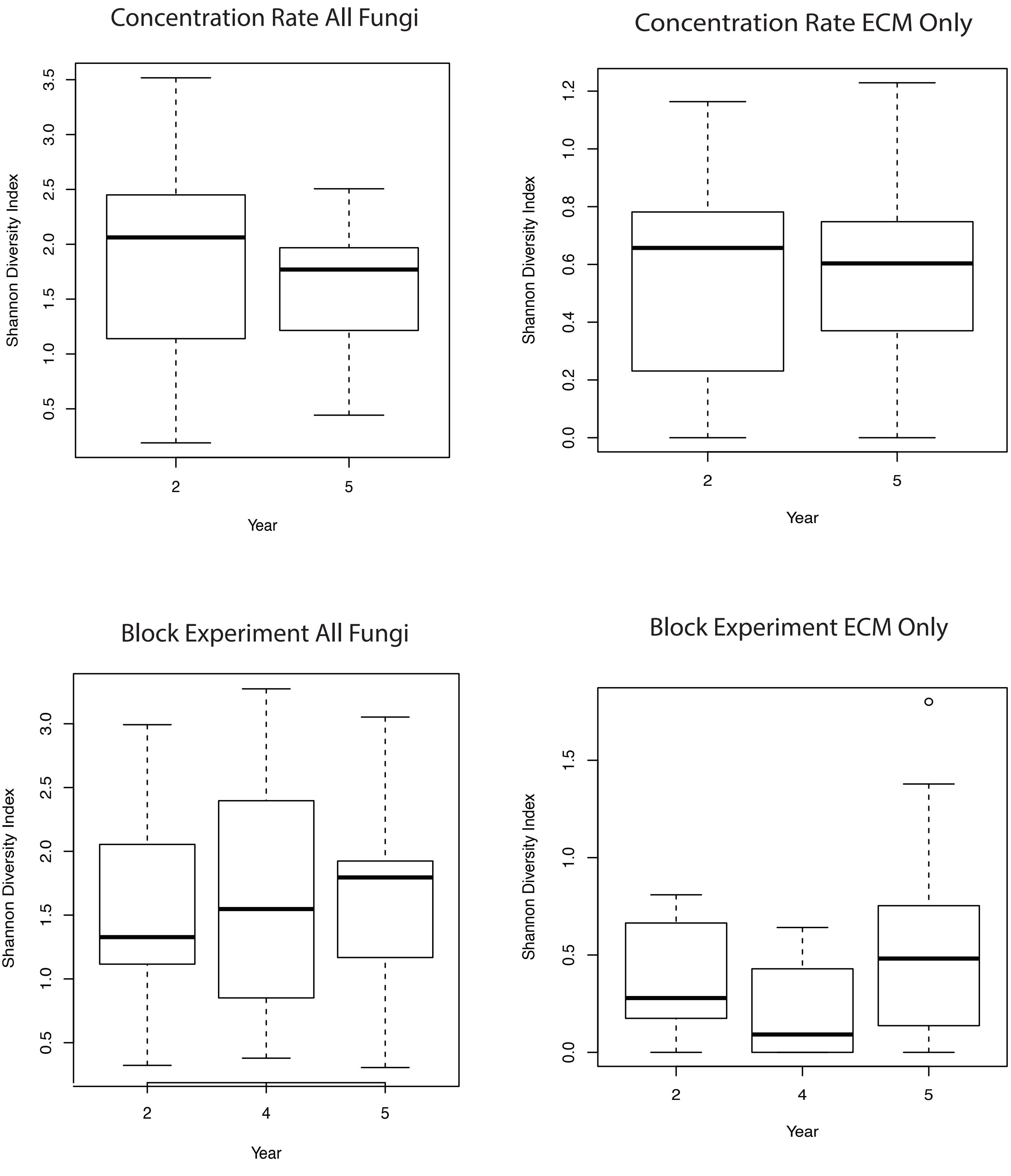

Fig. S1: Shannon Diversity box plots of the concentration rate experiment and block experiment for the All Fungi datasets and the ECM Only datasets. These anaylses were only conducted for site Tift because it was the only site where multiple years of data collection could be obtained.
